# Chromosomal dynamics in space and time: evolutionary history of *Mycetophylax* ants across past climatic changes in the Brazilian Atlantic coast

**DOI:** 10.1038/s41598-019-55135-5

**Published:** 2019-12-11

**Authors:** Ricardo Micolino, Maykon Passos Cristiano, Natália Martins Travenzoli, Denilce Meneses Lopes, Danon Clemes Cardoso

**Affiliations:** 1 Departamento de Genética, Universidade Federal do Paraná (UFPR), Curitiba, PR Brazil; 2 Departamento de Biodiversidade, Evolução e Meio Ambiente, Universidade Federal de Ouro Preto (UFOP), Ouro Preto, MG Brazil; 3 Departamento de Biologial Geral, Universidade Federal de Viçosa (UFV), Viçosa, MG Brazil

**Keywords:** Cytogenetics, Evolution, Phylogenetics

## Abstract

Fungus-farming ants of the genus *Mycetophylax* exhibit intra and interspecific chromosome variability, which makes them suitable for testing hypotheses about possible chromosomal rearrangements that endure lineage diversification. We combined cytogenetic and molecular data from *Mycetophylax* populations from coastal environments to trace the evolutionary history of the clade in light of chromosomal changes under a historical and geographic context. Our cytogenetic analyses revealed chromosomal differences within and among species. *M*. *morschi* exhibited three distinct karyotypes and considerable variability in the localization of 45S rDNA clusters. The molecular phylogeny was congruent with our cytogenetic findings. Biogeographical and divergence time dating analyses estimated that the most recent common ancestor of *Mycetophylax* would have originated at about 30 Ma in an area including the Amazon and Southern Grasslands, and several dispersion and vicariance events may have occurred before the colonization of the Brazilian Atlantic coast. Diversification of the psammophilous *Mycetophylax* first took place in the Middle Miocene (*ca*. 18–10 Ma) in the South Atlantic coast, while “*M*. *morschi*” lineages diversified during the Pliocene-Pleistocene transition (*ca*. 3–2 Ma) through founder-event dispersal for the Northern coastal regions. Psammophilous *Mycetophylax* diversification fits into the major global climatic events that have had a direct impact on the changes in sea level as well as deep ecological impact throughout South America. We assume therefore that putative chromosomal rearrangements correlated with increased ecological stress during the past climatic transitions could have intensified and/or accompanied the divergence of the psammophilous *Mycetophylax*. We further reiterate that “*M*. *morschi*” comprises a complex of at least three well-defined lineages, and we emphasize the role of this integrative approach for the identification and delimitation of evolutionary lineages.

## Introduction

Ants (Hymenoptera: Formicidae) exhibit astonishing ecological success that has been attributed to their social organization as well as the ability to associate with other organisms, *e*.*g*., fungi^1,2^. Another remarkable feature of the ants is their impressive karyotype variation that ranges from 2*n* = 2 to 2*n* = 120^3,4^. Such exceptional variations in the chromosomal number may indicate that ants underwent speciation processes concomitantly with chromosomal rearrangements (CRs). Chromosome numbers tend to be stable within a species, so a potential polymorphism of this trait into a population or lineage becomes an important step toward intraspecific delimitation^5,6^. It has been proposed that CRs may promote speciation through reproductive isolation^7–10^, although it remains a source of debate, increasingly studies agree^11–13^. In fact, observations of ant karyotypic diversity were determinant for the proposition of the Minimum Interaction Theory, which suggests that Robertsonian (Rb) fissions are the main driving force in long-term chromosome evolution and are effective at minimizing genetic risks and increasing the potential for genetic divergence^14–16^.

**Figure 1:**
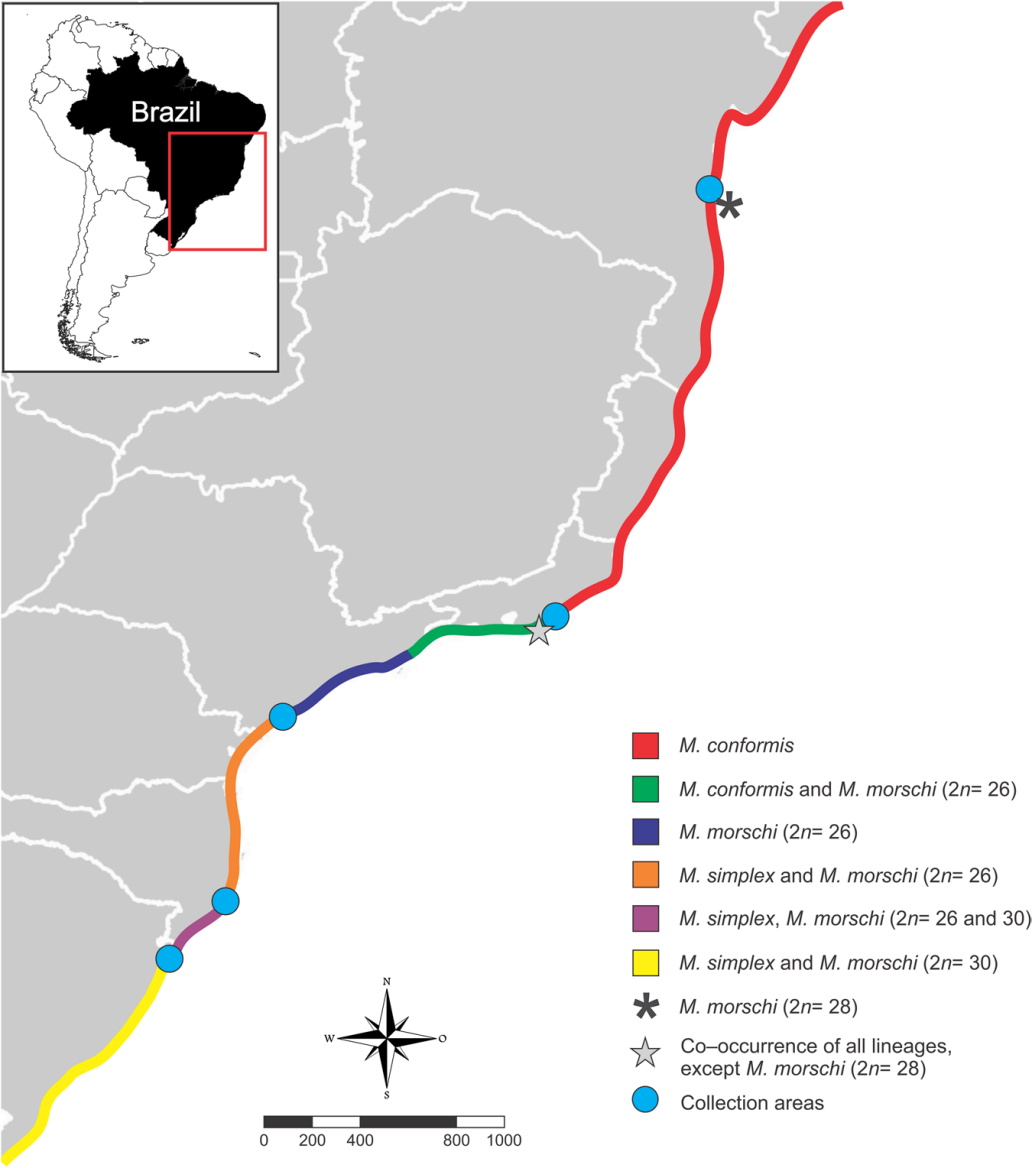
Map of geographic distribution and sampling localities of *Mycetophylax* populations along Brazilian Atlantic coast. The legends of the colors and symbols are embedded in the figure. The scale bar is represented in kilometers. Adapted from Cardoso *et al*.^60^.

In contrast, in a population-based cytogenetic study with fungus-farming ants of the genus *Mycetophylax*, the role of centric fusions in the karyotypic diversification of those ant lineages was proposed^17^. *Mycetophylax* was formerly composed of three endemic species of coastal sand dune environments, known as Restinga^18,19^: *M*. *conformis* Mayr, 1884; *M*. *morschi* Emery, 1888; and *M*. *simplex* Emery, 1888 (see Fig. 1 for details of occurrence areas). Differences in both the number and structure of their chromosomes were found, including intraspecific variation within *M*. *morschi*, showing populations with two distinct karyotypes (2*n* = 26 and 2*n* = 30). It has therefore been suggested that *M*. *morschi* comprises a complex of cryptic species or even different lineages that have been subjected to consecutive CRs^17^. It is noteworthy that through phylogenetic analyses, members of the former *Cyphomyrmex strigatus* group, including *Mycetosoritis asper* and *Mycetosoritis clorindae*, were shown to form a well-defined clade with strong statistical support along with *Mycetophylax sensu stricto*. Thus, these species were transferred to the genus *Mycetophylax*, which currently comprises 21 species^20^.

The integration of cytogenetic data, phylogenetic trees, and molecular dating considerably improved the findings on evolutionary history within lineages as well as providing implications that perhaps could not be found independently. However, few studies have combined such approaches, but what has emerged from this integration are interesting and even unexpected results^21–24^. Divergence times and phylogenetic relationships can be estimated simultaneously under a Bayesian inference. A method for divergence time estimation and fossil calibration using a stochastic branching process and relaxed-clock model in a Bayesian framework was recently developed^25,26^. The Fossilized Birth-Death (FBD) process has the advantage of assuming that fossils and molecular sequences from extant species comprise the same diversification process^25–27^. On the other hand, under a molecular cytogenetic approach, such as Fluorescence *in situ* Hybridization (FISH) chromosome mapping, useful diagnostic characters for morphologically conservative species may be provided as variations in the number and/or position of ribosomal DNA (rDNA) clusters or CR indications given by changes in chromosome number. Hence, the use of FISH data has become an important tool for describing and delimiting new taxa, especially for insects^28–30^. In this way, we can accurately test the assumptions made previously on the likely chromosomal evolution within the *Mycetophylax* lineages.

Additionally, the endemism of the three former *Mycetophylax* species to the Atlantic coastal environments remains a peculiar and attractive feature since the correlation between chromosomal and genetic variability needs to be examined in the context of geographic distributions of the species^31,32^. The Brazilian Atlantic coastal area (often referred to as the Restinga ecosystem) can be physically and biologically defined as coastal plains of marine sedimentary origin^33^ that were subjected to ecological and geological stress during the major climatic transitions associated eustatic change^34,35^. Global past climate changes have certainly had biogeographic impacts due to the effects of alterations in temperature and moisture on species^36,37^. Ancestral range estimation contributes to a better understanding of species distributions over evolutionary time scales, and it is possible to infer dispersion and/or vicariance events from previous climatic and/or geological events in a given geographic area^38^. The Restinga ecosystem is a coastal environment within the Atlantic Forest biome that is floristically and geomorphologically heterogeneous. It is composed of a range of organisms that have intrinsically adapted to adverse conditions^39^. These habitats have been deeply influenced by the long-term effects of climate change that may have contributed to species diversification due to dynamism. Therefore, we hypothesized that *Mycetophylax* populations may have diversified through chromosomal changes triggered by the climatic events that have continuously affected their habitat.

In order to test the assumptions raised above, we correlated cytogenetic, molecular, and biogeographic data from the additional *Mycetophylax* species with the phylogenetic tree of the fungus-farming ants, and under a historical and geographical context, we aimed to describe the evolutionary relationships and chromosomal changes that raised the karyotype observed today. In addition, with this integrative approach, we aimed to decipher the taxonomic status of *M*. *morschi*, which is currently considered a single species, but which certainly represents a complex of independent evolutionary lineages.

## Results

### Karyotype determination and FISH mapping

**Figure 2:**
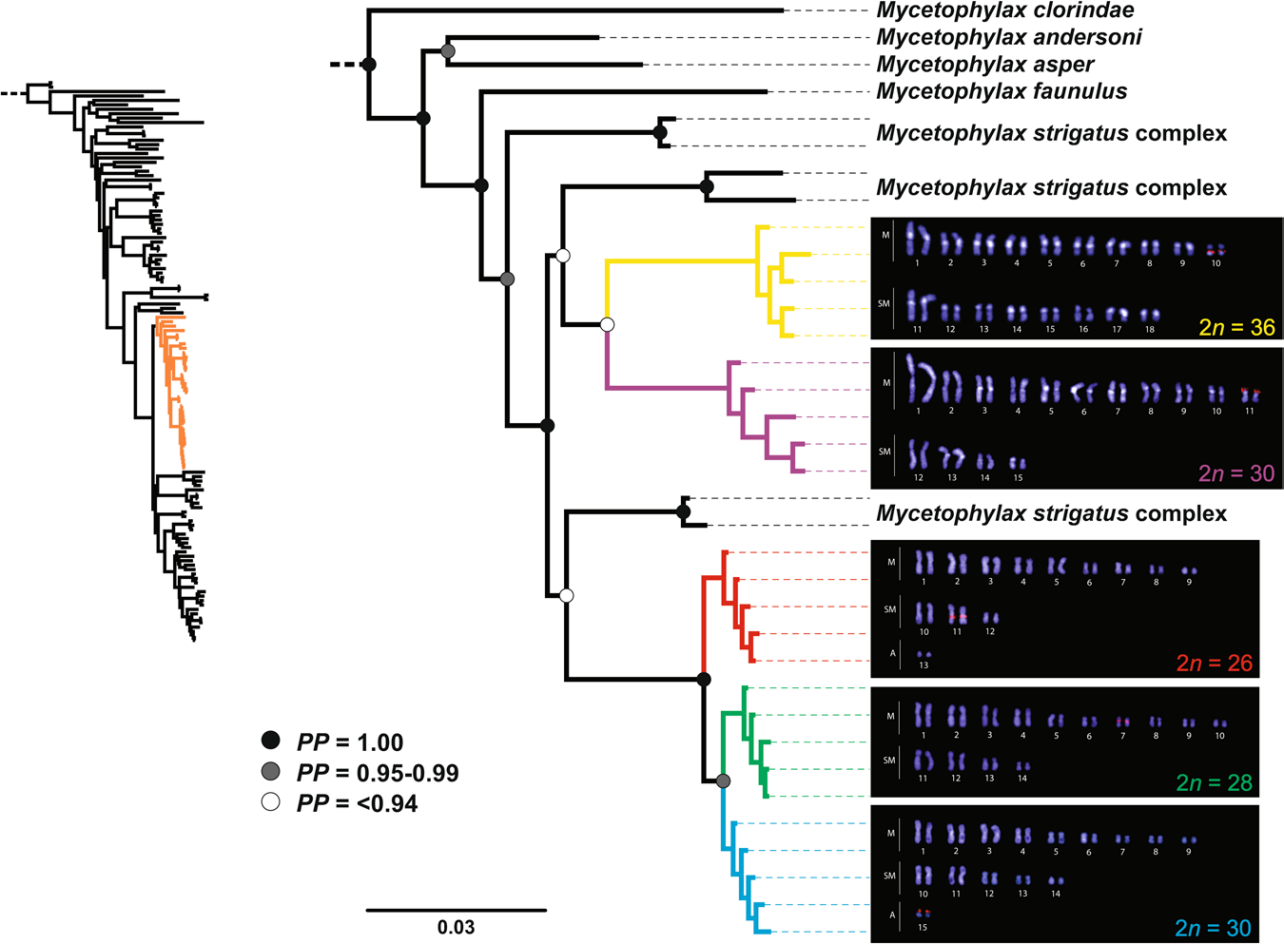
Pruned phylogenetic tree from *Mycetophylax* fungus-farming ants based on a Bayesian analysis of five nuclear protein-coding genes and their DAPI-stained karyotypes showing the FISH mapping of the 18S rDNA probe (in red). *M*. *simplex* shown in yellow branches and karyotype 2*n* = 36. *M*. *conformis* shown in purple branches and karyotype 2*n* = 30. *M*. *morschi* (lineage A) shown in red branches and karyotype 2*n* = 26. *M*. *morschi* (lineage B) shown in green branches and karyotype 2*n* = 28. *M*. *morschi* (lineage C) shown in blue branches and karyotype 2*n* = 30. In the karyotypes images: (M) metacentric, (SM) submetacentric, and (A) acrocentric chromosomes.

The detailed cytogenetic analysis with *Mycetophylax* populations revealed the presence of intra- and inter-specific karyotype variations related to the number of diploid chromosomes (2*n*), the karyotypic formula (KF), and the fundamental number (FN). FISH signals with the 18S rDNA probe showed a stable number of rDNA clusters, denoting only one pair of chromosomes, but its localization and position along the chromosome differed between karyotypes (Fig. 2). The distribution pattern of the TTAGG_(6)_ telomeric repeat was restricted to the terminals of both chromosome arms in all karyotypes and intercalated stronger and weaker signals at random (Fig. S1). No signals for interstitial telomeric sites (ITS) were detected.

*Mycetophylax morschi* surprisingly exhibited three distinct cytotypes (*i*.*e*., organisms considered to be from the same species but differing in both number and morphology of the chromosomes) instead of two, and thus, it was divided into three lineages, as follows:

#### Lineage A

Colonies from the southern (Torres/RS) and southeast (Cabo Frio/RJ and Ilha Comprida/SP) Brazilian coast presented 2*n* = 26 chromosomes. This karyotype is composed of nine metacentric, three submetacentric, and one acrocentric chromosome pairs; the karyotype formula is KF = 9 M + 3SM + 1 A; and the fundamental number (FN) is 50. The rDNA clusters are located in the pericentromeric region of the long arms of submetacentric chromosome pair 11 (Fig. 2).

#### Lineage B

Colonies from the northeast Brazilian coast (Ilhéus/BA) presented 2*n* = 28 chromosomes. This unexpected karyotype is composed of ten metacentric and four submetacentric chromosome pairs with a karyotype formula of KF = 9 M + 5SM and an FN of 56. The rDNA clusters are located in the pericentromeric region of the short arms of submetacentric chromosome pair 7 (Fig. 2). Note that, unlike the others, this lineage does not present acrocentric chromosomes.

#### Lineage C

Colonies of both localities of the southern Brazilian coast (Torres/RS and Araranguá/SC) presented 2*n* = 30 chromosomes. This karyotype is composed of nine metacentric, five submetacentric, and one acrocentric chromosome pairs with a karyotype formula of KF = 9 M + 5SM + 1 A and an FN of 58. The rDNA clusters are located in the terminal region of the short arms of the acrocentric chromosome pair (Fig. 2). For the first time, the presence of two cytotypes of “*M*. *morschi*” was recorded at the same geographic area. It is therefore conclusive that they live sympatrically.

#### Mycetophylax conformis

All colonies of *M*. *conformis* presented 2*n* = 30 chromosomes. This karyotype is composed of eleven metacentric and four submetacentric chromosome pairs with a karyotype formula of KF = 11 M + 4SM and an FN of 60. The rDNA clusters are located in the terminal region of the short arms of metacentric chromosome pair 11 (Fig. 2).

#### Mycetophylax simplex

All colonies of *M*. *simplex* presented 2*n* = 36 chromosomes. This karyotype is composed of ten metacentric and eight submetacentric chromosome pairs with a karyotype formula of KF = 10 M + 8SM and an FN of 72. The rDNA clusters are located in the pericentromeric region of the long arms of metacentric chromosome pair 10 (Fig. 2).

### Molecular phylogeny

Our phylogenetic analyses were based on Bayesian Inference (BI), which exhibited strong statistical support for most nodes (Figs. 2 and S2). We use the aligned dataset provided by Sosa-Calvo *et al*.^20^ and inserted additional *Mycetophylax* sequences obtained in this work. The phylogenetic tree generated showed three well-supported clades of “*M*. *morschi*”: the first one corresponded to the cytotype 2*n* = 26 (lineage A) (Bayesian posterior probability (PP) = 1.0), and the others diversifying from it formed a sister group of cytotypes 2*n* = 28 (lineage B) and 2*n* = 30 (lineage C) (PP = 0.96) (Fig. 2). These three lineages were grouped with unknown species of the former *Cyphomyrmex strigatus* complex, composing a clade with intriguing weak support. *M*. *simplex* and *M*. *conformis* fell in a sister group (PP = 0.75), which, together with another unknown species of the former *C*. *strigatus* complex, formed a major clade with “*M*. *morschi*” lineages and the unknown species from the *C*. *strigatus* complex (PP = 1.0) (Fig. 2). This major clade will henceforth be called “psammophilous *Mycetophylax*”.

### FBD-based divergence dating

**Figure 3:**
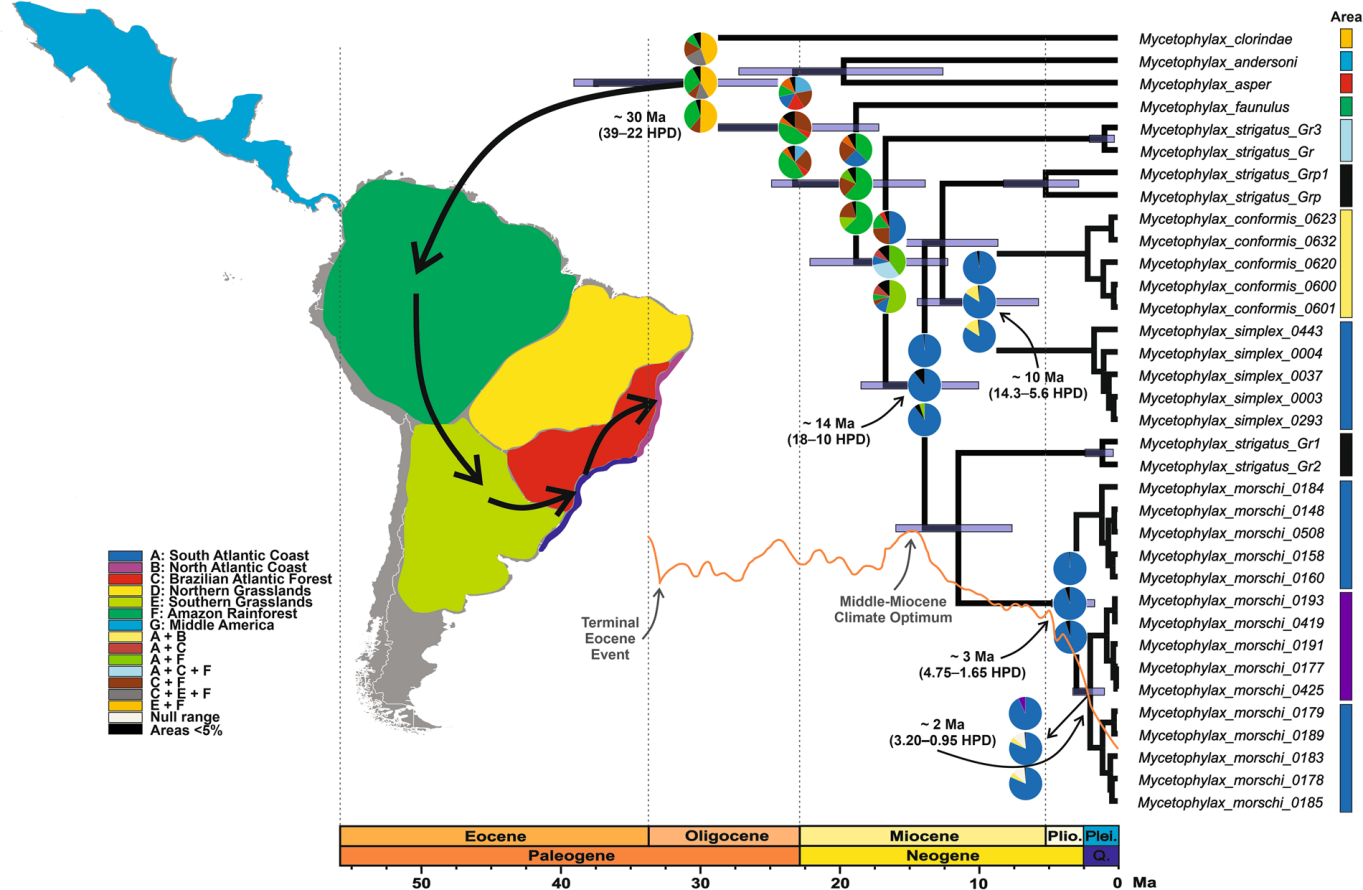
Phylogenetic tree based on Fossilized Birth-Death process representing the divergence time estimates along with ancestral range estimates based on three alternative biogeographic model. The arrows on the map represent a possible route of the *Mycetophylax* ants along the colonization of the Brazilian Atlantic coast. Pie charts showing (**a**) “BioGeoBEARS” analyses (model selected: BAYAREALIKE + j) on the top, (**b**) the BayArea model on the middle, and (**c**) the Bayesian Binary MCMC model on the bottom. The pie charts at the nodes represent 95% confidence intervals of the relative frequencies of the ancestral range optimizations across the restricted tree of the genus *Mycetophylax*. The horizontal blue bars at the nodes represent the 95% highest posterior density (HPD) intervals of the estimated node ages. The numbers on the main nodes represent the mean ages of lineage divergence as well as the confidence intervals. The scale axis bar represents million years ago (Ma). Q.: Quaternary, Plio.: Pliocene, and Plei.: Pleistocene. The orange curve represents temperature fluctuations during the past ∼35 Ma, as depicted by Zachos *et al*.^34^.

The FBD-based divergence dating analysis recovered the stem-group age (*i*.*e*., the earliest possible origin) of fungus-farming ants as 64.6 million years ago (Ma) and the crown-group age (*i*.*e*., the latest possible origin) as 61.9 Ma (95% highest posterior density interval, HPD = 76–49 Ma) (Fig. S3). The stem- and crown-group ages for the *Mycetophylax* clade were reconstructed as 37.4 and 29.9 Ma, respectively (HPD = 39–22 Ma), and psammophilous *Mycetophylax* emerged at about 14 Ma (HPD = 18–10 Ma) (Fig. 3). Divergence age estimates of ((*M*. *conformis* + *M*. *simplex*) + (*strigatus* complex “01”)) and ((“*M*. *morschi*” lineages) + (*strigatus* complex “02”)) indicated that they diverged almost simultaneously at 12.5 Ma (HPD = 17.2–8.6 Ma) and 11.4 Ma (HPD = 15.9–7.6 Ma), respectively. While *M*. *conformis* and *M*. *simplex* split at about 10 Ma (HPD = 14.3–5.6 Ma), the “*M*. *morschi*” lineages diversified more recently: lineage A diverged from lineages B + C at ~3 Ma (HPD = 4.75–1.65 Ma), while lineages B + C diverged at ~2 Ma (HPD = 3.2–0.95 Ma) (Fig. 3).

### Historical biogeography

The three independent estimates of the ancestral range showed similar results. In the BGB analysis, the BAYAREALIKE + j model yielded the best statistical fit for the data (AIC = 101.9; *p* = 0.06), although it had no significant difference to the model without the founder-event speciation parameter (Table S1). All models pointed out that the most recent common ancestor (MRCA) of *Mycetophylax* would have originated in an area including the Amazon and Southern Grasslands (BGB: *p* = 0.4, BayArea: *p* = 0.43, and BBM: *p* = 0.5) (Fig. 3). Most of the nodes estimated dispersal while vicariance was recovered in a few nodes, including the split of the “*M*. *morschi*” lineages.

The colonization of the coastal regions probably involved major dispersal events that would have directed the *Mycetophylax* ancestors to the Atlantic Forest from the Amazon and subsequently spread to the South Coast region at about 17 Ma (BGB: *p* = 0.37, BayArea: *p* = 0.59, and BBM: *p* = 0.62). All models strongly estimated the ancestral range of all psammophilous *Mycetophylax*, including the remaining ants from the “*strigatus* complex”, which apparently lived on the Atlantic coast of Southern Brazil and later scattered farther north in both coastal and rainforest regions, including the Amazon (BGB: *p* = 0.98, BayArea: *p* = 0.89, and BBM: *p* = 0.89) (Fig. 3). No dispersion events were recovered, primarily indicating diversification within the area or sympatric range inheritance.

The ancestral range of *M*. *conformis* and *M*. *simplex* was estimated as the South Atlantic Coast for all models (BGB: *p* = 0.97, BayArea: *p* = 0.84, and BBM: *p* = 0.84). Thus, *M*. *simplex* inherited the ancestral range, whereas *M*. *conformis* colonized the North Atlantic coast via founder-event dispersal. Likewise, the “*M*. *morschi*” lineages would have inhabited the South Atlantic Coast (BGB: *p* = 0.99, BayArea: *p* = 0.95, and BBM: *p* = 0.95), and their diversification may have been sympatrical, as no dispersal event was estimated. Finally, lineages B and C of “*M*. *morschi*” would have diverged through two dispersion events and one of vicariance. Lineage C could have colonized the North Atlantic Coast via founder-event dispersal (BGB: *p* = 0.93, BayArea: *p* = 0.81 and BBM: *p* = 0.81) (Fig. 3).

### Chromosome evolution

**Figure 4:**
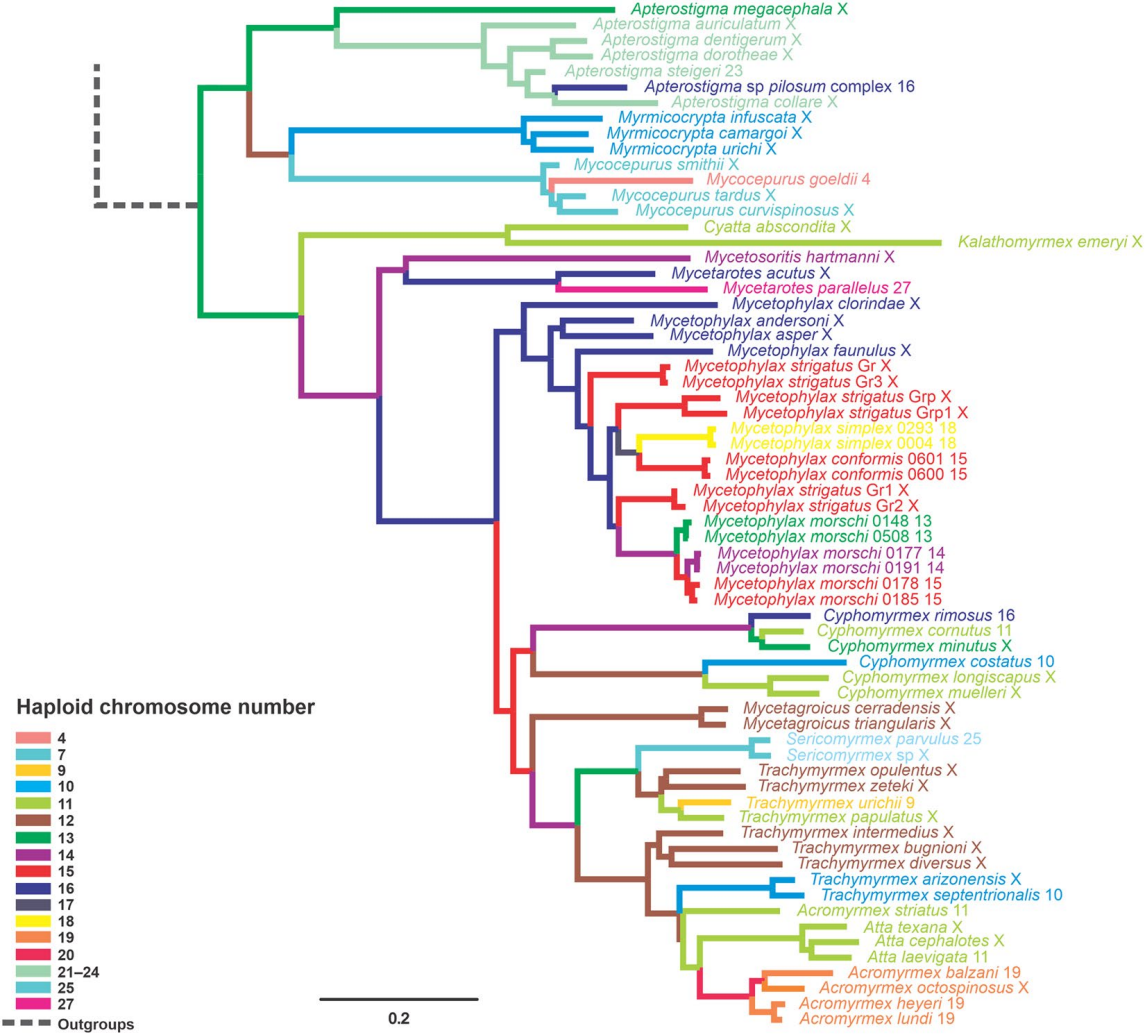
Chromosome number evolution and inferred ancestral chromosome state in the phylogenetic tree of fungus-farming ants from ChromEvol results based on Bayesian inference, chosen because it provides posterior probabilities as a statistical parameter. The numbers at the tips are the known haploid chromosome numbers of species, while “X” represents unknown number. The various colors on the branches of the tree represent the base haploid chromosome number for each node, given in the legend of the figure.

Our ancestral chromosome numbers reconstruction suggests that the best model to support chromosome evolution is linear gain, loss, and duplication. This model infers that the rates of gain (fission) and loss (fusion) of a chromosome are constant and depend linearly on the current number of chromosomes. The estimated rate parameters in the best model are shown in Table S2. The number of haploid chromosomes estimated in the MRCA of the genus *Mycetophylax* ranged from *n* = 15 to *n* = 17 between the ML and BI analyses (Figs. 4 and S4). In the psammophilous *Mycetophylax* clade, the most likely haploid numbers were *n* = 15 (ML) and *n* = 16 (BI; PP = 0.20). The ancestral karyotype number of the sister clades *M*. *conformis* and *M*. *simplex* was *n* = 16 (ML) and *n* = 17 (BI; PP = 0.25). Likewise, the likely chromosomal ancestry of the “*M*. *morschi*” lineages was *n* = 14 for both approaches (PP = 0.50 for BI). On the other hand, the ancestral karyotype number of the clade containing “*M*. *morschi*” lineages B and C was *n* = 14 (ML) and *n* = 15 (BI; PP = 0.51) (Figs. 4 and S4).

## Discussion

### Chromosome evolution and diversification of psammophilous *Mycetophylax*

Our study involved extensive sampling of psammophilous *Mycetophylax* populations in order to evaluate the level of diversification of these lineages on their geographic distribution along the Brazilian Atlantic coast and to deeply investigate the mechanisms of chromosome evolution in a phylogenetic context. Previous analyses with psammophilous *Mycetophylax* showed remarkable chromosomal polymorphisms among populations^17^, which provoked us to seek additional knowledge about their evolutionary relationships. Our data corroborate the previous karyotypic findings^17^ on the species *M*. *conformis* and *M*. *simplex*, both in terms of the number and structure of their chromosomes. Likewise, our data support the findings of dissimilar karyotypes within *M*. *morschi* populations with the unexpected discovery of a new karyotype (2*n* = 28) in a geographic area previously unknown to this species, the northeast Brazilian Atlantic coast. No universal rule seems to govern karyotype evolution, but a chromosomal variation between closely related lineages, such as psammophilous *Mycetophylax*, strongly suggests the role of CRs in the lineage diversification. In fungus-farming ants, few species have had their karyotypes described so far (45 out of 245), but available data show extensive variation among species (2*n* = 8 to 2*n* = 50)^4^. This aspect, which appears to be typical of Formicidae, also corresponds to the clade of the fungus-farming ants and may be the outcome of diverse CRs, such as Rb fusion/fission.

In an attempt to partially understand the speciation history of the psammophilous *Mycetophylax*, we integrated cytogenetic and phylogenetic data. Our cytogenetic information has provided some evidence that psammophilous *Mycetophylax* comprises distinct and new evolutionary lineages, since different chromosomal numbers eventually do not match during meiosis, acting as post-zygotic barriers. Besides, as rDNA is considered one of the most conservative fraction from the eukaryotic genome^40,41^, variations in the location and chromosome bearer of the rDNA clusters, as observed in the karyotypes analyzed here, may be related to the differentiation and divergence of natural populations^42^. In fact, rDNA clusters are even considered hotspots of chromosomal changes and lineage diversification in mice^43^. Here, we found that all of the *Mycetophylax* karyotypes and cytotypes showed only two signals of 45S rDNA but at distinct locations and homologue pairs suggesting that changes in ribossomal cluster appears to be related to the diversification of these ants. Some chromosomal mechanisms have been suggested to explain such mobility of rDNA clusters within sister lineages, including ectopic recombination^44^, transposition^45^, and centric fission^46^. The fact that there is no variation in the number of these clusters can be attributed to homologous recombination between repetitive sequences dispersed throughout genome (*i*.*e*., ectopic recombination) or more likely to transposition of these sequences into arbitrary position and chromosomes. However, we cannot state any further inferences, only that the intraspecific chromosomal diversity in *M*. *morschi* gives us further indications that these lineages may indeed be considered distinct species.

The application of molecular phylogenetics on the study of karyotype evolution has enabled us to deduce the direction of changes that give rise to chromosome variation in the most objective way^47,48^. For example, just because two species of a genus share the same chromosome number does not mean that they are phylogenetically closer to each other than two species with different chromosome numbers^5^. However, the base chromosome number (*i*.*e*., the haploid number present in the initial population of a monophyletic clade) may be directly related to the chromosomal variability inside that clade and its sister group^5^. Our phylogenetic reconstruction is congruent with our cytogenetic findings showing that the *Mycetophylax* cytotypes are phylogenetically close. The lineage A diverged prior to the other “*M*. *morschi*” lineages, which would imply that 2*n* = 26 (*n* = 13) was probably the ancestral karyotype. However, our results on chromosome evolution reconstructed its ancestral karyotype as 2*n* = 28 (*n* = 14), whereas it was 2*n* = 32 (*n* = 16) for the clade *M*. *conformis* + *M*. *simplex*. Previous estimates have shown slightly contrasting results, with an ancestral diploid number for “*M*. *morschi*” lineages (*n* = 15) and the clade *M*. *conformis* + *M*. *simplex* (*n* = 17), leading the authors to propose fusion as the main mechanism of chromosome evolution in *Mycetophylax*^17^.

Our FISH-based comparative approach indicates that is more likely that both Rb fissions and fusions contributed to the karyotypic diversification in psammophilous *Mycetophylax* and similarly in Formicidae rather than a larger role for Rb fissions, as has been proposed^14–16^. In fact, specifying which chromosomes are involved in such rearrangements is a difficult task, especially since large-scale CRs can modify karyotypes in a striking way. For example, a karyotypic reduction observed in *Cucumis* species (from 2*n* = 24 to 2*n* = 14) involved at least 59 CRs, including five fusions, four translocations, and 50 inversions^49^. Not least, the presence of only a single pair of chromosomes bearing the 45S rDNA clusters in fungus-farming ants appears to be uniform. Taking into account the phylogenetic relationships of fungus-farming ants from the basal species *Mycocepurus goeldii*^50^ and cross referencing our data with the most derived species of leafcutter ants from *Acromyrmex* and *Atta* genera^51,52^, we noticed that there are only a pair of chromosomes bearing the 45S rDNA cluster, which would imply that this chromosomal feature may be plesiomorphic.

Fusions can be directly observed using telomeric probes. After *in tandem* fusions, the canonical telomeric sequences may remain in the interstitial chromosome regions, serving as clues of fusion and inversions involving the telomeres. Telomeric sequences can either be eliminated or not be eliminated by chromosome breakage, and when complete elimination does not occur, interstitial telomeric sites (ITS) can be observed^53^. Considering the fusion hypothesis proposed by Cardoso *et al*.^17^ as the mechanism shaping *Mycetophylax* karyotypes, signals for ITS would be expected, but we did not find any evidence for ITS, suggesting that if tandem fusion did take place the telomeric regions would have been lost. Lastly, we added more Formicidae species containing the TTAGG as the ending motif on their chromosomes, and thus we reinforce the hypothesis that this sequence could have been the ancestral motif of the ant telomeres^54,55^.

### Evolutionary history of psammophilous *Mycetophylax* under a cytogenetic perspective

Since the beginning of the Cenozoic (*ca*. 65 Ma), the Earth’s biota has experienced both rapid and gradual climate changes^34,35^ that have had profound impacts on species diversification and composition^56^. Indeed, the major climate transitions has triggered higher rates of species turnover, and the Paleocene-Eocene Thermal Maximum “PETM” (*ca*. 55 Ma), the Terminal Eocene Event “TEE” (*ca*. 34 Ma), Middle Miocene Climatic Optimum “MMCO” (*ca*. 16–14 Ma), and Pliocene-Pleistocene intervals (from *ca*. 3 Ma) have been reported as striking events for mammals^56^. Coincidentally, our time-calibrated phylogenetic reconstruction suggested that the basal divergence of the genus *Mycetophylax* took place in the Eocene-Oligocene boundary (*ca*. 38–22 Ma), while psammophilous *Mycetophylax* diverged into the Middle Miocene (*ca*. 18–10 Ma) and thereafter diversified into the existing lineages. Both inferred ages fit with one of these major events mentioned above, which makes them relevant to other taxa than mammals, including here invertebrates.

The fungus-farming ants originated about 60 Ma in South America^57^, likely during the post-extinction event recovery period and shortly before the PETM. After that, an abrupt drop in temperature began at around 52 Ma and continued to the end of the Eocene (*i*.*e*., TEE)^34^ that had left hallmarks characterized by cooler and more seasonal climates, increase topographic heterogeneity, and persistent phylogenetic and ecologic diversification of lineages^58^. It was suggested therefore that this global cooling event spurred both fungus-farming ants’ lineages diversification and ant-fungus coevolution^57^. Likewise, this assumption agrees with our estimated age for diversification of the genus *Mycetophylax* in the Miocene (*ca*. 23–5.3 Ma). The Miocene was a climatically dynamic interval from Cenozoic, when long-term global climatic cooling was punctuated by extreme climatic optima (*i*.*e*., MMCO)^59^. After this period of global warming — the warmest one in the last 24 Ma^34^— a climate transition associated with major Antarctic ice sheet expansion and global cooling began, the so-called Middle Miocene Glaciation “MMG”^59^, which drastically extended to the Pleistocene glacial intervals. As these global climate oscillations were directly associated with marine transgressions and regressions^35^, the evolutionary history of psammophilous *Mycetophylax* on sand dunes could plausibly be related to the heterogeneity of the Brazilian Atlantic coast as well to their topographic and climatic gradients.

The basal split between *M*. *conformis* and *M*. *simplex* took place in the Middle to Late Miocene, *ca*. 10 Ma (HPD = 14.3–5.6 Ma), corresponding to the initial cooling period (*i*.*e*., MMG) and a consequent drop in sea levels reaching near to the current levels^35^. These species are morphologically distinguishable^18^ and are not found to inhabit the same coastline area, except in Cabo Frio, Rio de Janeiro^60^. *M*. *conformis* is further distributed in the north of the Atlantic coast with the southern limit of its occurrence at about 24°S, while *M*. *simplex* occurs from the southern coast of São Paulo (~25°S) to the border of southern Brazil (~34°S)^60^. Our ancestral range estimates place the species ancestor as having inhabited the South Atlantic Coast, and we identified a major dispersal event. The putative biogeographic scenario would be that *M*. *simplex* inherited the ancestral range, while *M*. *conformis* would have colonized the North Atlantic Coast via founder-event dispersal. Founder-event speciation refers to changes in the lineage distribution during split (cladogenesis), where one daughter lineage inherits the ancestral range and the other lineage “jumps” to a new area^61,62^. Considering the current contrasting geographic range and respective karyotypes of *M*. *simplex* and *M*. *conformis*, the fixation of different CRs either by genetic drift or geographic isolation could have been triggered by the recurrent climatic events that continuously changed the environment, leading CRs to play some role in lineage diversification. Moreover, relict populations of *M*. *simplex* living sympatrically with *M*. *conformis* could further indicate that species were sympatric in the past and that during successive Pleistocene climatic transitions they could have become allopatric majority. Phylogeographic analyses with *M*. *simplex* populations corroborate this, as it was identified that the species maintained a stable population size until about 75,000 years ago when it underwent a gradual demographic expansion^63^.

Continuous climate changes have been more abrupt since the Early Pliocene leading to extreme periods of glaciation (most temperate regions) and aridification (tropical regions) later in the Pleistocene^58^. Particularly in South America, these fluctuations were responsible for chorological changes that expanded and contracted the distribution areas of taxa, communities, and biomes^36^. Indeed, increasing climatic oscillations during the past 3 Ma have driven many range changes for a range of taxa, and higher speciation rates were identified during the Pliocene and Pleistocene^64,65^, which may well be associated with chromosomal changes promoting lineage diversification and potential reproductive isolation^32,66^. The chromosomal diversity observed in psammophilous *Mycetophylax* is in agreement with this scenario, especially considering “*M*. *morschi*” lineages. The divergence of these lineages was recovered at the Pliocene-Pleistocene boundary (*ca*. 3.6–1.8 Ma). Historical biogeography analyses have estimated the ancestral range as the South Atlantic Coast with no dispersion events, suggesting that at least the initial diversification of this group may have occurred sympatrically. Interestingly, it has been argued that sympatric sister taxa have a higher average karyotypic diversity than allopatric sister taxa^67^. Populations that have multiple accumulated CRs can persist more easily in secondary sympatry than populations whose karyotypes are insufficiently divergent, thus leading sympatric populations to reproductive divergence^7,8,67^. On the other hand, the split of “*M*. *morschi*” lineages B and C would have involved both dispersion and vicariance events, allowing a possible colonization route of diversification to be traced.

The Pliocene-Pleistocene intervals had large-scale effects on habitat change, including the Brazilian coast, which was remarkably influenced by the sea level^35,58^. Sea level fluctuations related to climate change and geological disturbances would have dramatically increased over these periods to −100 m from current sea level^35,58^, thus allowed the opening of new previously unexploited regions by psammophilous taxa, including *Mycetophylax* ants. A direct consequence of this severe drop in sea level during the glacial periods would be the emergence of the Brazilian continental shelf^68^. This displacement of the southeastern South American coastline would have reached hundreds of kilometers to the east, enabling suitable climatic conditions for forest-adapted species^68^ and presumably extending the geographic range of psammophilous taxa to areas previously flooded by the sea. Populations of “*M*. *morschi*” subjected to putative CRs would have been confronted with adaptively adverse conditions as a result of climatic and ecological stress during colonization, favoring CRs that would increase adaptation to these conditions whenever such conditions arose^10,11^. This pattern of chromosomal changes and local adaptation has been reported for *Anopheles gambiae*^11^ and supports the diversity and karyotypic evolution in the psammophilous *Mycetophylax* clade. Additionally, the perspective of CRs fixation in isolated and/or peripheral founder populations through genetic drift and inbreeding is raised by many of the chromosomal speciation models^9–11^. In this way, the northernmost dispersion of the Brazilian Atlantic coast by the psammophilous *Mycetophylax* where molded by several historical events coupled with chromosomal changes, likely Rb fissions and fusions that shaped the karyotypes and lineage differentiation.

In conclusion, our integrative approach combining cytogenetic and molecular data has once again proved to be effective in supporting systematics and taxonomic research. We emphasize that the lack of cytogenetic data is still a hindrance to better application of these approaches. Further, we shed light in the cryptic diversity within “*M*. *morschi*” and, as previously suggested^17^, should be treated as phylogenetically isolated lineages or even as distinct species as they form well-resolved clades. The entire genus *Mycetophylax* has undergone systematic and taxonomic changes over the years, reinforcing the need for taxonomic revision. Psammophilous *Mycetophylax* represents a cytogenetically diverse group, showing great variation in terms of the number and morphology of the chromosomes and the localization of highly conserved sequences, such as the 45S rDNA cluster. Fossil-calibrated molecular dating suggests that the genus *Mycetophylax* probably originated at the Eocene-Oligocene boundary in the Amazon and/or Southern Grasslands, while psammophilous *Mycetophylax* emerged in the Middle Miocene in the South Atlantic coast, and most diversification occurred during subsequent major climatic events in the sand dune areas of the Brazilian Atlantic Forest biome. Putative CRs in the context of global climatic events and subsequent ecological stress could have somehow triggered and accompanied the diversification of the lineages in this highly dynamic environment.

## Materials and Methods

### Biological material

Populations of *Mycetophylax* were previously collected throughout their area of geographic distribution along the Brazilian Atlantic coast (Fig. 1). Colonies of *M*. *morschi* were collected in coastal regions belonging to the following Brazilian cities: Torres/RS (29°22′00″S; 49°44′45″W), Araranguá/SC (28°54′37″S; 49°22′00″W), Ilha Comprida/SP (24°44′29″S; 47°32′12″W), Cabo Frio/RJ (22°54′37″S; 42°02′40″W), and Ilhéus/BA (14°29′60″S; 39°02′07″W). Colonies of *M*. *conformis* were collected from Cabo Frio/RJ (22°54′29″S; 42°02′15″W), while *M*. *simplex* colonies were collected from Araranguá/SC (28°56′57″S; 49°22′15″W) (Fig. 1). Each population collected consisted of at least five colonies. Subsequent to collection, the colonies were transported to and conditioned *in vivo* at the Laboratório de Genética Evolutiva e de Populações from Universidade Federal de Ouro Preto, Minas Gerais, Brazil to obtain pre-pupae larvae, as reported by Cardoso *et al*.^69^.

### Chromosome preparation and FISH

Mitotic metaphases were obtained by the method described by Imai *et al*.^14^ using brain ganglia of pre-pupae larvae through dissection in colchicine-hypotonic solution, following the adjusts described by Cardoso *et al*.^70^. Conventional giemsa staining was used to determine the chromosome number and morphology. We classified the chromosomes following the standard nomenclature for centromere position proposed by Levan *et al*.^71^ with the small modification on the term acrocentric referring to all the chromosomes with an arm ratio greater than 7.0. The arm ratio (*r*) is the ratio of the length of the long arm of a chromosome to that of the short arm. Thus, metacentric chromosomes (M) are those with *r* = 1.0–1.7, submetacentrics (SM) have *r* = 1.7–3.0, subtelocentrics (ST) have *r* = 3.0–7.0, and acrocentrics have values from 7.0 to above.

FISH experiments were performed according to previous descriptions^72^ with detailed modifications by Micolino *et al*.^73^. Highly conserved DNA repeat sequences (18S rDNA and TTAGG-telomeric motif) were used as probes. The TTAGG_(6)_ motif was directly labeled with Cy3 at the 5′ terminal during synthesis (SIGMA, ST. LOUIS, MO, USA). The 18S rDNA sequence was isolated from bee *Melipona quinquefasciata* and amplified by PCR using the primers 18SF (5′-GTCATATGCTTGTCTCAAAGA-3′) and 18SR (3′-TCTAATTTTTTCAAAGTAAACGC-5′)^74^, which corresponded to a 750 base pair (bp) segment. The PCR reaction consisted of initial denaturation for 3 min at 94 °C, 35 cycles of 1 min at 94 °C, 1 min at 55 °C, and 1 min at 72 °C, and final extension for 5 min at 72 °C. Metaphase chromosomes were incubated with RNAse (40 μg/mL) for 1 h at 37 °C. Thereafter, chromosomes were washed several times with saline solutions and then denatured in 70% formamide/2xSSC. The probes were hybridized with the chromosomes in 20 μL of the hybridization mix (200 ng of labeled probe, 50% formamide, 2xSSC, and 10% dextran sulfate 20xSSC) overnight. After this, metaphases were washed in 4xSSC/Tween, dehydrated in a series of alcoholic solutions (50%, 70%, and 100%, respectively) and assembled in antifade solution with DAPI (4′,6-diamidino-2-phenylindole) (DAPI FLUOROSHIELD, SIGMA-ALDRICH). Metaphase chromosomes were analyzed under an OLYMPUS BX53 epifluorescence microscope with OLYMPUS CELLSENS IMAGING software (OLYMPUS AMERICAN, INC., CENTER VALLEY, PA, USA), using WU (330–385 nm) and WG (510–550 nm) filters for DAPI and rhodamine, respectively. From 10 to 20 metaphases were analyzed per colony and the images were edited with ADOBE PHOTOSHOP CC software.

### DNA extraction, amplification, and sequencing

Genomic DNA extraction was performed from one ant worker per colony according to the standard CTAB/chloroform techniques^75^ with adaptations for ant extraction. We sequenced fragments of five nuclear protein-coding genes: *elongation factor 1-alpha-F1* (EF1α-F1), *elongation factor 1-alpha-F2* (EF1α-F2), *wingless* (Wg), *long-wavelength rhodopsin* (LW Rh), and *topoisomerase 1* (Top1) (GenBank accession numbers: MN745212-MN745286). The primers used to generate the sequence data are listed in Table S3. PCR was performed in a final volume of 25 μL which included 12.5 μL of Go*Taq* G2 Hot Start Colorless MasterMix (*Taq* DNA Polymerase, dNTPs, MgCl_2_, and buffer), 9.5 μL of Nuclease-Free Water, 1 μL of each pair of primers, and 1 μL of DNA, according to the manufacturer’s instructions (PROMEGA, MADISON, WI, USA). Amplifications reaction conditions and sequencing were based on the methodology outlined in previous studies^20,76,77^. The chromatogram quality was verified by a Phred quality score higher than 30, and then the sequences were manually trimmed in GENEIOUS R7^78^.

### Bayesian phylogenetic analysis

The concatenated dataset consisting of ~2.2 kbp (279 bp for EF1α-F1, 333 bp for Wg, 387 bp for LW Rh, 459 bp for EF1α-F2, and 669 bp for Top1) was added to the aligned dataset of Sosa-Calvo *et al*.^20^ and aligned by using the MEGA7^79^. Data were partitioned and modeled using PARTITIONFINDER 2^80^ under the Bayesian Information Criterion (BIC), greedy search scheme and with 15 input data blocks consisting of the first, second, and third codon positions of each of the five gene fragments. Thereafter, nine partitions were employed in independent Bayesian analyses using MRBAYES v3.2^81^. The standard nucleotide model was used for all partitions, and the nucleotide substitution models were variable for each partition (Table S4). Two simultaneous independent Monte Carlo Markov chain (MCMC) runs were performed with seven heated chains and one cold; the temperature was set at 0.05; and 10 million generations were performed with sampling every 1000 generations. The convergence among runs was verified by the average standard deviation of split frequencies that had to reach <0.01, and the appropriated burn-in was determined to be 10%. The tree-generated and posterior probabilities were visualized in FIGTREE v1.4^82^.

### Divergence time estimates

For divergence time analyses, we used BEAST v2.5^83^ under the Fossilized Birth-Death (FBD) model^26^ using an uncorrelated, log normal relaxed clock model to describe the branch-specific substitution rates^84^. The FBD model has become the most appropriate way to calibrate divergence time estimates when the calibration dates represented fossil occurrence times^26^. Accordingly, we integrated the occurrence times of 13 sets of Myrmicinae ant species fossils^85^ into the tree prior to impose a time structure on the tree and to calibrate the analysis to absolute time. We calibrated internal nodes with minimum-age constraints using Myrmicinae amber fossils (Table S5). The root age was set at 98.6 Ma based on the estimated age for the subfamily Myrmicinae^77^. We set the nucleotide substitution model as GTR + I + G for all genes. Independent MCMC analyses were run for 60 million generations, sampling every 1000 generations. Runs were evaluated using TRACER v1.7^86^ with effective sample size (ESS) values for all parameters over 200. The first 20% of the sampled tree topologies from the analyses were discarded as burn-in, and the remaining trees were summarized in TREEANNOTATOR v2.5. Before this, all fossils were removed from the tree using the FullToExtantTreeConverter tool (implemented in BEAUTI v2.5). The generated trees and credible intervals were visualized in both FIGTREE v1.4^82^ and ICYTREE^87^.

### Biogeographical analysis and ancestral range estimation

Biogeographical reconstructions were performed using a phylogenetic tree constructed only with *Mycetophylax* species plus their basal relatives: *Mycetarotes*, *Mycetosoritis*, *Kalathomyrmex* and *Cyatta* species. It should be stressed that some specimens of the “*strigatus* group” were pruned from the tree as they are unknown lineages with unknown geographic ranges as well. We ran the phylogenetic tree using BEAST v2.5^83^ with 25 million generations, sampling every 1000 generations, and a final burn-in of 10%. For this, we used a Yule speciation process, an uncorrelated, log normal relaxed clock model, and GTR + I + G for all genes. We characterized seven geographic ranges by the Neotropics that corresponded to the species distributions used: South Atlantic Coast (A); North Atlantic Coast (B); Brazilian Atlantic Forest (C); Northern Grasslands, including the Caatinga and Cerrado biomes (D); Southern Grasslands, including Chaco and Pampas biomes (E); Amazon Rainforest (F); and Middle America, covering all of Central America and Southern USA (G). The species distributions were taken from www.antmaps.org ^88^.

We used the RASP v4.1^89^ software package to estimate the likely ancestral ranges of *Mycetophylax*. RASP implements the major model-based approaches currently used, which enables comparisons to be made. Three approaches were employed for such estimates. The first one was carried out with the BioGeoBEARS (BGB)^90^ to compare alternative biogeographic models. BGB can implement six specific models: the dispersal-extinction-cladogenesis model (DEC)^91^, a likelihood version of the dispersal-vicariance analysis (DIVALIKE), and the Bayesian inference of historical biogeography for discrete areas (BAYAREALIKE); and all three models can also be run with the founder-event speciation parameter (+j)^38,61^. The model also implements the rate of “dispersal” (range expansion) and “extinction” (range contraction) along the internal branches of the phylogeny^38,61^. In order to analyze the connectivity between the areas, we set up dispersion multipliers so that adjacent areas received a dispersion multiplier of 1 and non-adjacent areas received a value of 0.5. AIC results were used to assess the statistical significance of data-to-model fit.

The second approach used was BayArea^92^ as it re-calculates the ancestral state probabilities of each unit area with alternative burn-in values^89^. BayArea also incorporates geographic coordinate data, making it an attractive feature for biogeographic investigations. However, BayArea may need to be repeated several times to get a stable result^89^, so we carried out independent runs with 10 million generations each, setting different burn-in values. Lastly, the third approach used was the Bayesian Binary MCMC (BBM)^93^. BBM analysis was performed using an estimated model, F81 + G, with a null root distribution, and the MCMC was run for 10 million generations with sampling every 1000 generations.

### Ancestral chromosome number reconstruction

The chromosome evolution model and the reconstruction of ancestral chromosome numbers was recovered with CHROMEVOL v2.0^94,95^ using both the maximum likelihood (ML) and Bayesian approaches. The chromosome evolution models evaluated were implemented as specified by Cardoso *et al*.^17^. To avoid over-representing the OTUs, and as several karyotype data on fungus-farming ants are unavailable, we used a restricted phylogeny, comprising data with cytogenetic information and only two species per genus per clade, since some genera were paraphyletic. The phylogeny was generated under the same specifications cited above for the MRBAYES analyses. The chromosome models over the phylogeny estimated in Bayesian inference and its null hypotheses were analyzed with 10,000 simulations, and the model that best fit the data set was selected under the Akaike Information Criterion (AIC).

## Supplementary information

supplementary information
