# Supplementary material for: Chromosomal dynamics in space and time: evolutionary history of Mycetophylax ants across past climatic changes in the Brazilian Atlantic coast
Source: Sci Rep. 2019 Dec 11;9:18800. doi: 10.1038/s41598-019-55135-5 (PMC6906305; doi:10.1038/s41598-019-55135-5)
Supplement: Supplementary file 1 — supplementary information [file 41598_2019_55135_MOESM1_ESM.docx]

**Chromosomal dynamics in space and time: evolutionary history of *Mycetophylax* ants across past climatic changes in the Brazilian Atlantic coast**

Ricardo Micolino^1,2^, Maykon Passos Cristiano^2^, Natália Martins Travenzoli^3^, Denilce Meneses Lopes^3^ & Danon Clemes Cardoso^1,2,*^

^1^Departamento de Genética, Universidade Federal do Paraná (UFPR), Curitiba, PR, Brazil.

^2^Departamento de Biodiversidade, Evolução e Meio Ambiente, Universidade Federal de Ouro Preto (UFOP), Ouro Preto, MG, Brazil.

^3^Departamento de Biologial Geral, Universidade Federal de Viçosa (UFV), Viçosa, MG, Brazil.

Correspondence and requests for materials should be addressed to D.C.C. (email: [danon@ufop.edu.br](mailto:danon@ufop.edu.br))

**Appendix A. Supplementary material**

**Table S1.** Comparisons of likelihood values (LnL), dispersal (d) and extinction (e) rates, *P*-value compared to the models with and without event-founder dispersal (j), and the Akaike Information Criterion (AIC) scores from each of the analyses in “BioGeoBEARS”.

| Model | LnL | d | e | j | AIC |
| --- | --- | --- | --- | --- | --- |
| DEC | –63.79 | 5 | 2.13 | – | 132.5 |
| DEC+j | –59.23 | 5 | 3.8 | 0.077 | 126.5 |
| DIVALIKE | –66.5 | 5 | 3.90E–07 | – | 137.9 |
| DIVALIKE+j | –63.2 | 5 | 1.05 | 0.072 | 134.4 |
| BAYAREALIKE | –64.33 | 4.51 | 5 | – | 133.6 |
| BAYAREALIKE+j | –46.95 | 1.89 | 4.86 | 0.06 | 101.9 |

**Table S2.** The estimated rate parameters for the chromosome evolution model that best fit the data – the linear gain, loss and duplication model. Loss refers to chromosomal fusions, gain refers to chromosomal fissions and duplication refers to complete duplication of the genome (polyploidy). The total of events inferred throughout the karyotype evolution along the phylogenetic tree.

| Rate parameters |  |  | Total events |
| --- | --- | --- | --- |
| Loss | Constant (δ) | 11.58 |  |
|  | Linear (δ_1_) | 1.88 |  |
|  |  |  | 487.93 |
| Gain | Constant (λ) | 31.52 |  |
|  | Linear (λ_1_) | –0.74 |  |
|  |  |  | 276.06 |
| Duplication | Constant (ρ) | 1.12 |  |
|  |  |  | 12.33 |

**Table S3.** List of primers used for amplification of the nuclear genes *EF1α-F1*, *EF1α-F2*, *Wg*, *LW Rh* and *Top1* in fungus-farming ants of the genus *Mycetophylax*.

| Gene region | Primer | Sequence 5′ to 3′ | Source |
| --- | --- | --- | --- |
| *EF1a-F1* | 1424F | GCGCCKGCGGCTCTCACCACCGAGG | Brady *et al.*^1^ |
|  | 1829R | GGAAGGCCTCGACGCACATMGG | Brady *et al.*^1^ |
| *EF1a-F2* | 557F | GAACGTGAACGTGGTATYACSAT | Brady *et al.*^1^ |
|  | 1118R | TTACCTGAAGGGGAAGACGRAG | Brady *et al.*^1^ |
| *LW Rh* | LR143F | GACAAAGTKCCACCRGARATGCT | Ward & Downie^2^ |
|  | LR639ER | YTTACCGRTTCCATCCRAACA | Ward & Downie^2^ |
| *Wg* | wg578F | TGCACNGTGAARACYTGCTGGATGCG | Ward & Downie^2^ |
|  | wg1032R | ACYTCGCAGCACCARTGGAA | Abouheif & Wray^3^ |
| *Top1* | TP1339F | GARCAYAARGGACCKGTRTTYGCACC | Ward & Sumnicht^4^ |
|  | TP2192R | GARCARCARCCYACDGTRTCHGCYTG | Ward & Sumnicht^4^ |

**Table S4.** The 15 partitions and models identified by PartitionFinder 2 and used in the Bayesian analyses of the concatenated dataset.

| Partition | Data blocks | Best model |
| --- | --- | --- |
| p1 | EF1aF1 pos1 | TRN+I |
| p2 | EF1aF1 pos2, EF1aF2 pos2 | F81+I |
| p3 | Wg pos3, EF1aF1 pos3 | GTR+I+G |
| p4 | Wg pos1, Wg pos2 | K80+I+G |
| p5 | Top1 pos2, LWRh pos1 | HKY+I+G |
| p6 | LWRh pos2 | GTR+I+G |
| p7 | LWRh pos3 | K80+I+G |
| p8 | Top1 pos1, EF1aF2 pos1 | GTR+I+G |
| p9 | Top1 pos3, EF1aF2 pos3 | TRNEF+I+G |

**Table S5.** List of a priori age distributions applied to 13 fossil calibration points for BEAST divergence dating analysis.

| Taxon (stem-group) | Offset  (in Ma) | Location | Source |
| --- | --- | --- | --- |
| *Myrmica* spp. | 42 | Baltic and Saxonian ambers | Radchenko *et al.*^5^ |
| *Tetramorium* spp. | 42 | Baltic and Saxonian ambers | Dlussky & Rasnitsyn^6^ |
| *Pristomyrmex* spp. | 42 | Late Eocene amber | Dlussky & Radchenko^7^ |
| *Pogonomyrmex fossilis* | 34 | Florissant Formation | Carpenter^8^ |
| *Pheidole* spp. | 34 | Florissant Formation | Carpenter^8^ |
| *Cataulacus* spp. | 30 | Sicilian amber | Emery^9^ |
| *Cephalotes atratus* | 15 | Dominican amber | De Andrade & Baroni Urbani^10^ |
| *Crematogaster acuta* | 15 | Dominican amber | Blaimer^11^ |
| *Strumigenys ambatrix* | 15 | Dominican amber | Baroni Urbani & De Andrade^11^ |
| *Apterostigma electropilosum* | 15 | Dominican amber | Schultz^12^ |
| *Cyphomyrmex maya* | 15 | Dominican amber | De Andrade^13^ |
| *Cyphomyrmex taino* | 15 | Dominican amber | De Andrade^13^ |
| *Trachymyrmex primaevus* | 15 | Dominican amber | Baroni Urbani^14^ |

**Supplementary Figures**


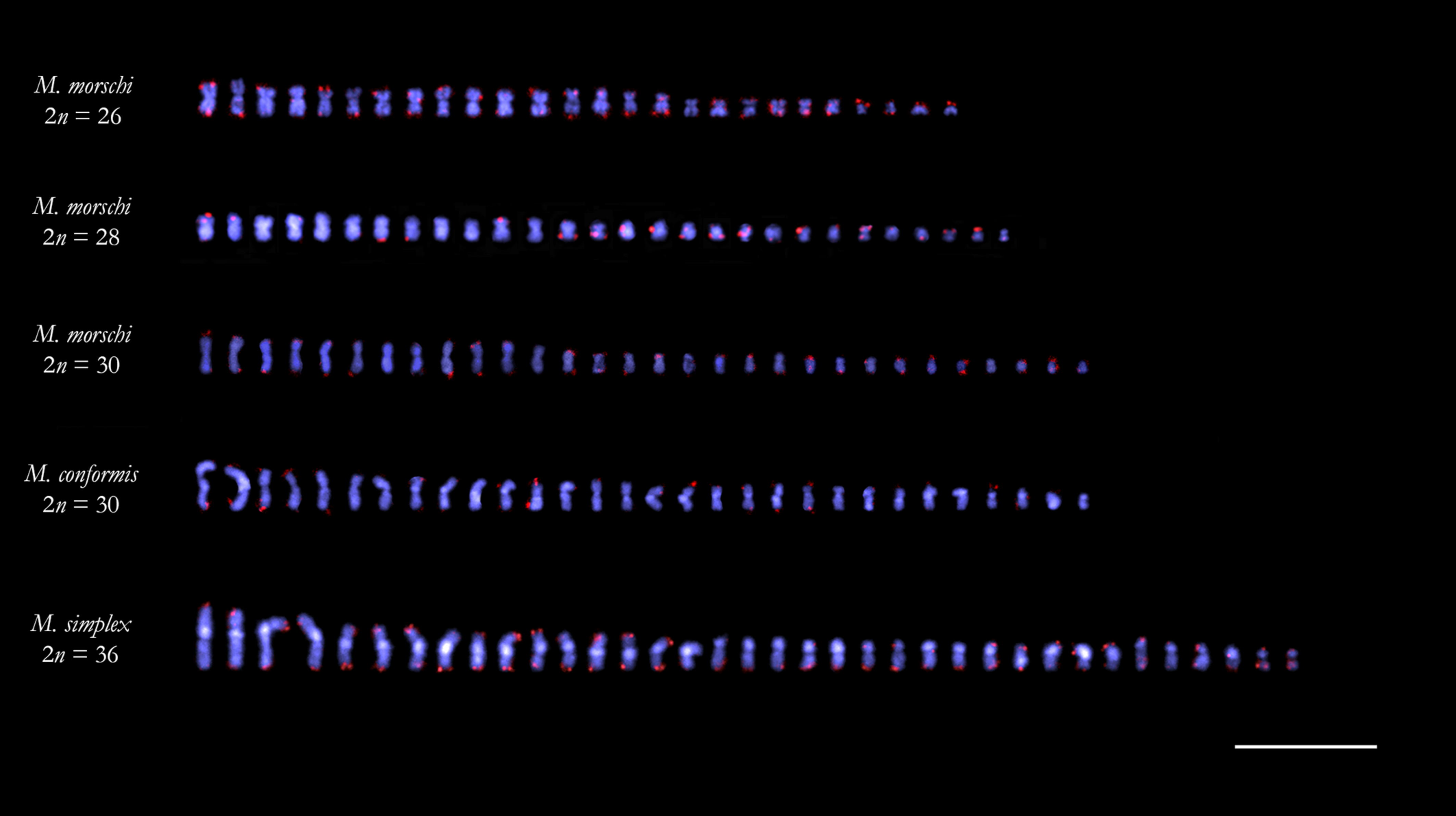


**Figure S1.** FISH mapping of TTAGG_(6)_ telomeric probe (in red) in DAPI-stained karyotypes from psammophilous *Mycetophylax*. a) *M. morschi* (2*n* = 26); b) *M. morschi* (2*n* = 28); c) *M. morschi* (2*n* = 30); d) *M. conformis* (2*n* = 30); and e) *M. simplex* (2*n* = 36). Scale bar = 5 µm.

**

**

**Figure S2.** Phylogeny of fungus-farming ants based on a Bayesian analysis of five nuclear protein-coding genes plus *Mycetophylax* lineages analyzed here, as follows: *M. simplex* (yellow); *M. conformis* (purple); “*M. morschi*” lineage A (red); “*M. morschi*” lineage B (green); and “*M. morschi*” lineage C (blue). Black dots on branches represent a Bayesian posterior probability of 100, while gray dots represent a Bayesian posterior probability of 95–99. Outgroups are represented by the dotted gray line.





**Figure S3.** FBD-based phylogenetic tree showing divergence time estimates of the fungus-farming ants. The horizontal blue bars at the nodes represent the 95% highest posterior density (HPD) intervals of the estimated node ages. The scale axis bar represents million years ago (Ma).

**

**

**Figure S4.** Chromosome number evolution and inferred ancestral chromosome state in the fungus-farming ants from ChromEvol results based on maximum likelihood (ML). The numbers at the tips are the known haploid chromosome numbers of species, while “X” represents unknown numbers. The various colors on the branches of the tree represent the base haploid chromosome number for each node, given in the legend of the figure.

**References**

1. Brady, S. G., Schultz, T. R., Fisher, B. L. & Ward, P. S. Evaluating alternative hypotheses for the early evolution and diversification of ants. *Proc. Natl. Acad. Sci. USA* **103**, 18172–18177 (2006). <https://doi.org/10.1073/pnas.0605858103>

2. Ward, P. S. & Downie, D. The ant subfamily Pseudomyrmecinae (Hymenoptera: Formicidae): phylogeny and evolution of big-eyed arboreal ants. *Syst. Entomol.* **30**, 310–335 (2005). <https://doi.org/10.1111/j.1365-3113.2004.00281.x>

3. Abouheif, E. & Wray, G. A. Evolution of the gene network underlying wing polyphenism in ants. *Science* **297**, 249–252 (2002). <https://doi.org/10.1126/science.1071468>

4. Ward, P. S. & Sumnicht, T. P. Molecular and morphological evidence for three sympatric species of *Leptanilla* (Hymenoptera: Formicidae) on the Greek Island of Rhodes. *Myrmecol. News* **17**, 5–11 (2012).

5. Radchenko, A. G., Dlussky, G. & Elmes, G. W. The ants of the genus *Myrmica* (Hymenoptera: Formicidae) from Baltic and Saxonian amber (Late Eocene). *J. Paleontol.* **81**, 1494–1501 (2007). <https://doi.org/10.1666/05-066.1>

6. Dlussky, G. M. & Rasnitsyn, A. P. Ants (Insecta: Vespida: Formicidae) in the Upper Eocene amber of central and Eastern Europe. *Paleontol. J.* **43**, 1024–1042 (2009). <https://doi.org/10.1134/S0031030109090056>

7. Dlussky, G. M. & Radchenko, A. G. *Pristomyrmex rasnitsyni* sp. n., the first known fossil species of the ant genus *Pristomyrmex* Mayr (Hymenoptera: Formicidae) from the Late Eocene Danish amber. *Russian Entomol. J.* **20**, 251–254 (2011).

8. Carpenter, F. M. The fossil ants of North America*. Bull. Mus. Comp. Zool.* **70**, 1–66 (1930).

9. Emery, C. Le formiche dell’ambra Siciliana nel Museo Mineralogico dell’Universita di Bologna. *Mem. R. Accad. Sci. Ist. Bologna* **5**, 141–165 (1891).

10. De Andrade, M. L. & Baroni Urbani, C. Diversity and adaptation in the ant genus *Cephalotes*, past and present. *Stutt. Beitr. Naturkd. B* **271**, 1–889 (1999).

11. Blaimer, B. B. Acrobat ants go global – origin, evolution and systematics of the genus *Crematogaster* (Hymenoptera: Formicidae). *Mol. Phylogenet. Evol.* **65**, 421–436 (2012). <https://doi.org/10.1016/j.ympev.2012.06.028>

12. Baroni Urbani, C. & De Andrade, M. L. First description of fossil Dacetini ants with a critical analysis of the current classification of the tribe (Amber Collection Stuttgart: Hymenoptera, Formicidae. VI: Dacetini). *Stutt. Beitr. Naturkd. B* **198**, 1–65 (1994).

13. Schultz, T. R. The fungus-growing ant genus *Apterostigma* in Dominican amber. *Mem. Am. Entomol. Inst.* **80**, 425–436 (2007).

14. De Andrade, M. L. First descriptions of two new amber species of *Cyphomyrmex* from Mexico and the Dominican Republic (Hymenoptera: Formicidae). *Beitr. Entomol.* **53**, 131–139 (2003).

11. Baroni Urbani, C. First description of fossil gardening ants (Amber Collection Stuttgart and Natural History Museum Basel; Hymenoptera: Formicidae. I: Attini). *Stutt. Beitr. Naturkd. B* **54**, 1–13 (1980).
